# Regulatory role of stem-loop structures in Faba bean necrotic yellows virus replication efficiency

**DOI:** 10.1128/spectrum.00941-25

**Published:** 2025-05-27

**Authors:** Muhammad Amir Qureshi, Muhammad Hassan Butt, Aamir Lal, Thuy T. B. Vo, Nattanong Bupi, Marjia Tabassum, Hyojin Im, Ahlim Lee, Amen Shamim, Sukchan Lee

**Affiliations:** 1 Department of Integrative Biotechnology, Sungkyunkwan University, Suwon, South Korea; 2 Changzhou Maternal and Child Health Care Hospital, Changzhou Medical Center, Nanjing Medical University, Changzhou, Jiangsu, China; 3 Department of Plant Medicals, College of Life Sciences, Andong National University 34920, Andong-si, Gyeongsangbuk-do, South Korea; 4 Division of Cancer Sciences, University of Manchester 5292, Manchester, England, United Kingdom; 5 Department of Computer Science, University of Agriculture 66724, Faisalabad, Punjab, Pakistan; Penn State College of Medicine, Hershey, Pennsylvania, USA

**Keywords:** multipartite virus, nanovirus, molecular dynamics simulation, common region-stem-loop, variation in neck region, expression analysis

## Abstract

Multipartite viruses package their genomic segments independently and primarily target plant hosts. A limited number of viruses are identified as targeting animals. The *Nanoviridae* is a family of multipartite single-stranded DNA (ssDNA) plant viruses that encapsidate approximately 1 kb ssDNA segments individually and transmit them via aphids in a circulative, non-propagative manner. Nanoviruses cause significant diseases in host plants, particularly in leguminous crops. Each segment of a nanovirus encodes an open reading frame that functions independently in the infection process. Additionally, each segment contains conserved inverted repeat sequences that create a stem-loop structure, along with a conserved nonanucleotide sequence known as “TAGTATTAC” located within the common region. This research analyzed the variations in the length of the stem-loop neck region of DNA-R segments of Faba bean necrotic yellows virus (FBNYV) and their implications using molecular dynamics (MD) simulations and experimental techniques. Although force field approximations and simulation time scales limit the quality of MD simulations, explicit solvent MD simulations successfully examined key characteristics of the stem-loop structure. The current study focused on the design of mutants based on variations in the neck region length of the stem-loop, construction of mutant infectious clones, and inoculation into the host plant, followed by expression patterns analysis of mutant stem-loop structures. The mutated stem-loop structures showed greater conformational stability compared to the wild type, and these variations in stability correspond to differences in FBNYV expression in the host plant. This finding establishes a framework for further structural and functional analyses of stem-loop structures of nanovirus.

**IMPORTANCE:**

Our stem-loop modification experiments in Faba bean necrotic yellows virus (FBNYV) highlight its critical role in viral replication and genome stability. Structural changes affected segment maintenance, emphasizing the need for precise stem-loop architecture for efficient replication. The findings provide novel insights into how FBNYV regulates genome integrity and segment functionality.

## INTRODUCTION

*Nanoviridae* represents a family of multipartite single-stranded DNA (ssDNA) plant viruses that encapsulate single-stranded DNA segments of around 1 kb. These viruses are transmitted through aphid vectors without replication, leading to significant diseases in leguminous crops and bananas (1–6). *Nanoviridae* is divided into two genera (Nanovirus and Babuvirus) according to their genomic structure and methods of transmission. Coconut foliar decay virus is an additional member of this family, yet it remains unassigned (5, 7). Nanoviruses exhibit nonenveloped structures characterized by icosahedral and spherical geometries, displaying *T* = 1 symmetry and measuring 18–19 nm in diameter. Nanoviruses consist of multipartite structures featuring 8–10 circular single-stranded DNA components, each about 1 kb in size (8–10). Each component is encapsulated into distinct capsids, with each segment serving a specific function (11).

**Fig 1:**
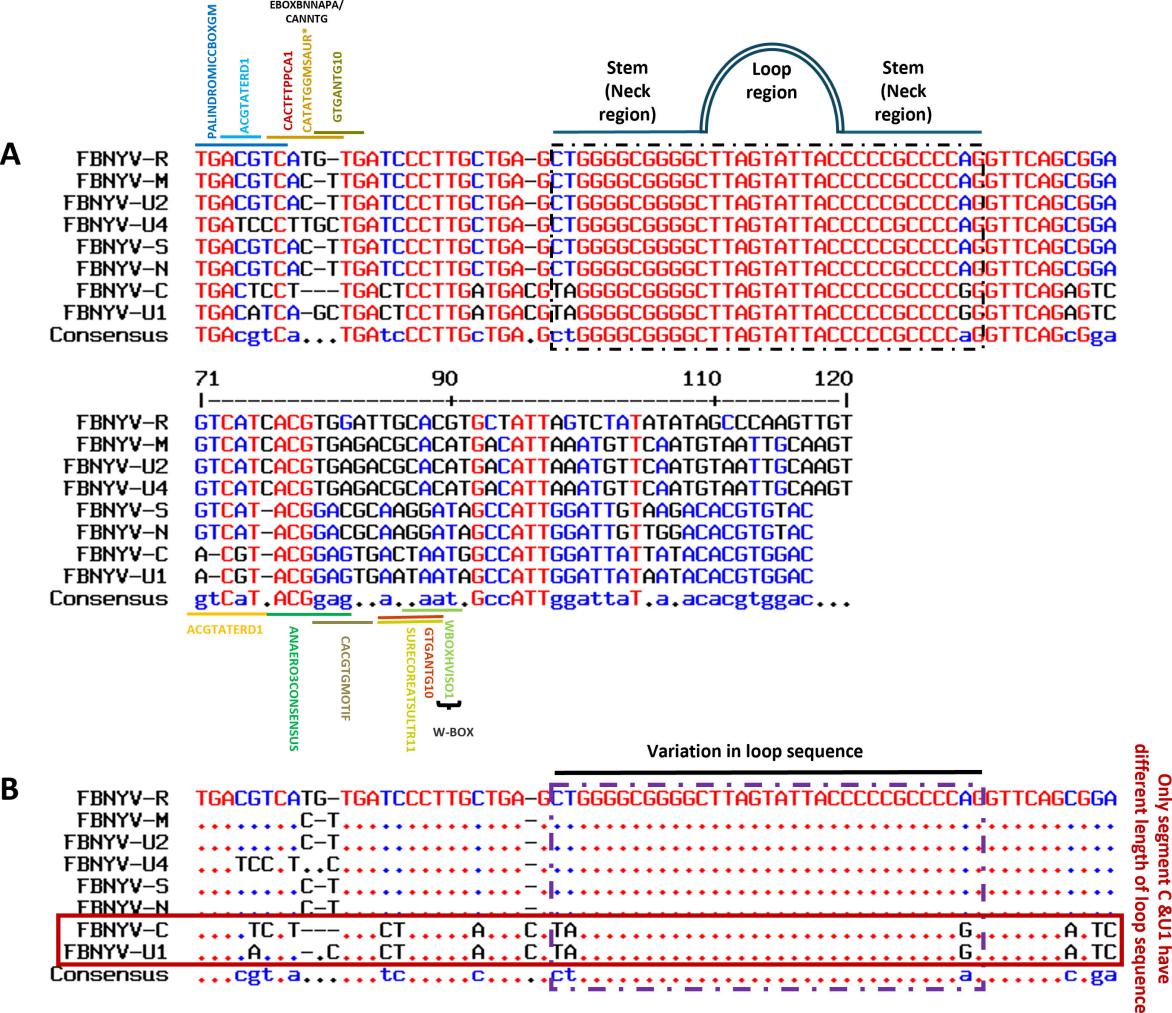
Comparison of the intergenic region (IR) across all segments of Faba bean necrotic yellows virus (FBNYV) and the variance in stem-loop length. (**A**) Alignment of IR across all segments in accordance with the conserved sequence of the stem-loop. Various motifs are observed both preceding and following the stem-loop, which shows the importance of the region. (**B**) Variance in the stem-loop sequences. The FBNYV-C and U1 segments possess a reduced neck region length of 9 nucleotides, in contrast to other segments, which exhibit a length of 11 nucleotides in the neck region.

Each segment of the nanovirus encodes an open reading frame (ORF) that fulfills a distinct function. Segment DNA R encodes the master replication initiator protein (12, 13), segment DNA C encodes the cell-cycle-link protein (14), segment M encodes the movement protein, DNA S encodes the capsid protein (15), and DNA N encodes the nuclear shuttle protein (8, 16). Numerous studies have examined the roles of U1, U2, and U4 in nanoviruses, as well as the satellite molecules associated with them; however, their functions remain mostly unclear (Fig. 1A). A recent study on the nanovirus Milk Vetch Dwarf Virus (MDV) found that the U2 component may produce a U2 protein that acts as a viral suppressor of RNA silencing, inhibiting single-stranded RNA and double-stranded RNA gene silencing (17).

The non-coding regions of each nanovirus segment conserve inverted repeat sequences, which are anticipated to form a stem-loop structure within a common region-stem-loop (CR-SL) and a nonanucleotide motif, “TAGTATTAC” (Fig. 1B). Nanoviruses replicate via a rolling-circle replication (RCR), with replication initiated by the viral Rep protein cleaving the conserved nonanucleotide sequence which is exclusive to CRESS-DNA viruses and is distinct from cellular DNA replication. Their diminutive, circular ssDNA genomes encode a replication-associated protein that commences RCR by nicking the viral strand at a designated origin site, thereby facilitating host polymerases in synthesizing a complementary double-stranded (ds) DNA intermediate. The Rep protein later enables the synthesis of new ssDNA genomes from this dsDNA replicative form. In contrast to cellular replication, nanoviruses need only one host machinery for nucleotide synthesis and elongation, and the process occurs in the nucleus, often alongside viral hijacking of host DNA repair and cell cycle pathways (8, 18, 19).

This study analyzed the impact of variations in the neck region of the stem-loop of Faba bean necrotic yellows virus (FBNYV) in the DNA R component on gene expression and viral replication. To delve deeper into the role of the stem-loop structures, molecular dynamic (MD) simulations were employed to evaluate the structural stability of these segments, providing insight into their functional significance. AMBER’s DNA OL15 force field and explicit Optimal Point Charge (OPC) water MD simulations were employed to analyze critical stability parameters, such as base-pair binding interactions, nucleotide fluctuations, cation binding, hydration, and binding free energies (20), to assess the impact of mutations on structural stability and their downstream effects on gene expression and viral replication. This integrated approach highlights the utility of combining structural bioinformatics and MD simulations with experimental virology to investigate the molecular mechanisms underlying nanovirus replication and gene regulation.

## RESULTS

### Sequence analysis and mutant aspects preparation in different composition

All genomic segments of FBNYV strain *EV1-93* contain conserved inverted repeat sequences to CR-SL with three short-repeated sequences, known as iterons (Fig. 1A). Key motifs and *cis* elements before and after the stem-loop structure can be seen in the intergenic regions (IRs). Accordingly, the IRs, particularly the CR-SL of all segments, were systematically analyzed, leading to the identification of segments with varying length stem-loop structures within various strains of FBNYV (Fig. S4). The alignment of all segments of FBNYV revealed that the CR-SL in the IR is the most conserved among segments. In DNA-U1 and DNA-C, variations were observed in the length of the stem-loop neck region, with nine nucleotide pairings, while DNA R, S, M, N, and U4 had 11 pairings (Fig. 1B). This variation may affect motif formation among segments.

**Fig 2:**
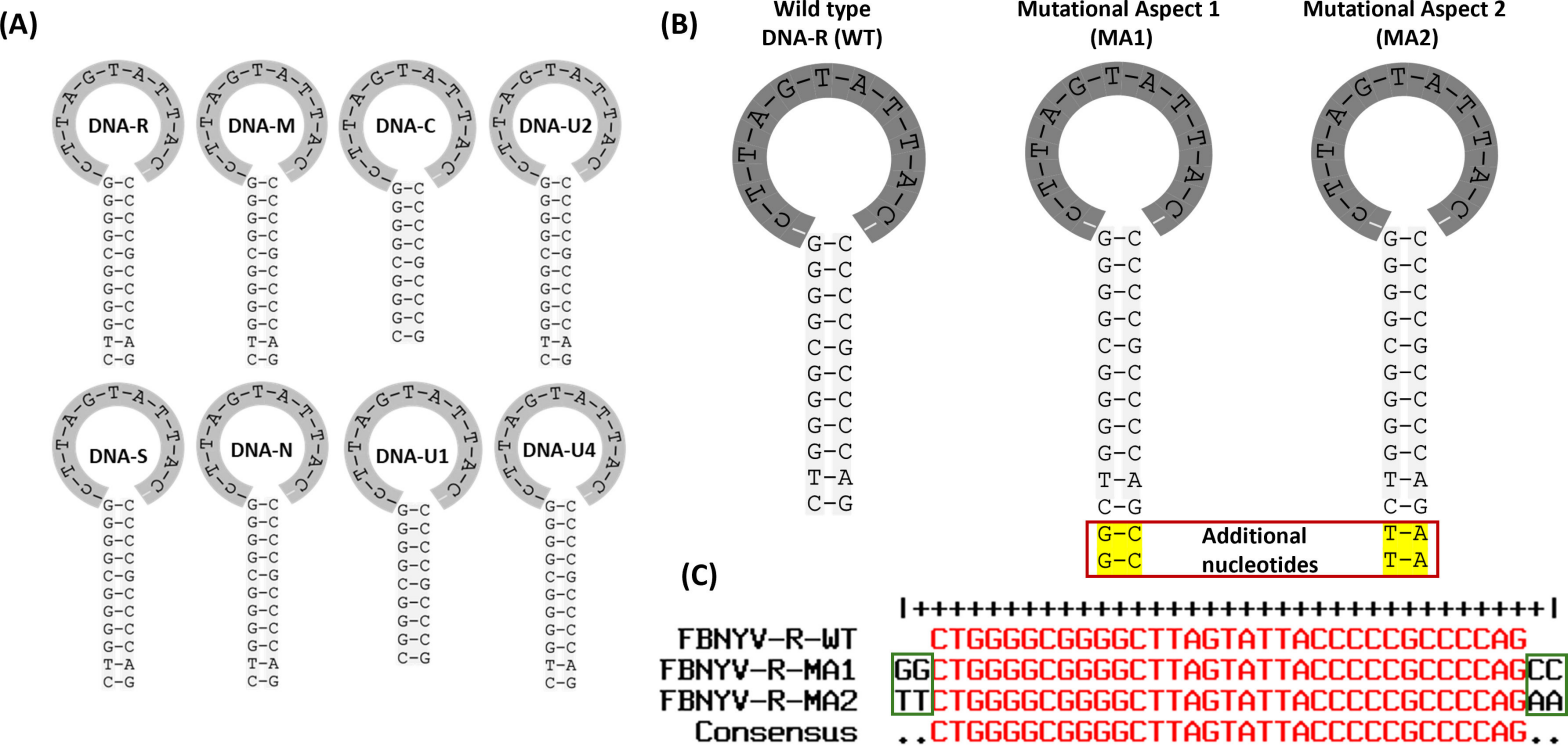
(**A**) Stem-loop structure of eight segments of FBNYV. (**B**) Stem-loop structure of DNA-R (WT, MA1 and MA2). (**C**) Sequence alignment of the stem-loop sequences f the WT, MA1, and MA2. Additional nucleotides in the neck region are highlighted in green.

Three aspects of FBNYV-DNA-R were analyzed, including wild type (WT) with 11 nucleotides in the neck region, mutational aspect 1 (MA1) with 13 nucleotides in the neck region with G and C pairing variation, and mutational aspect 2 (MA2) with 13 nucleotides in the neck region with T and A pairing variation (Fig. 2B). The stem-loop sequences from three aspects were aligned using the MultAlin tool, and the addition of mutant sequences was highlighted in both ends of the neck region (Fig. 2C).

### Molecular modeling of aspects of stem-loop sequence

**Fig 3:**
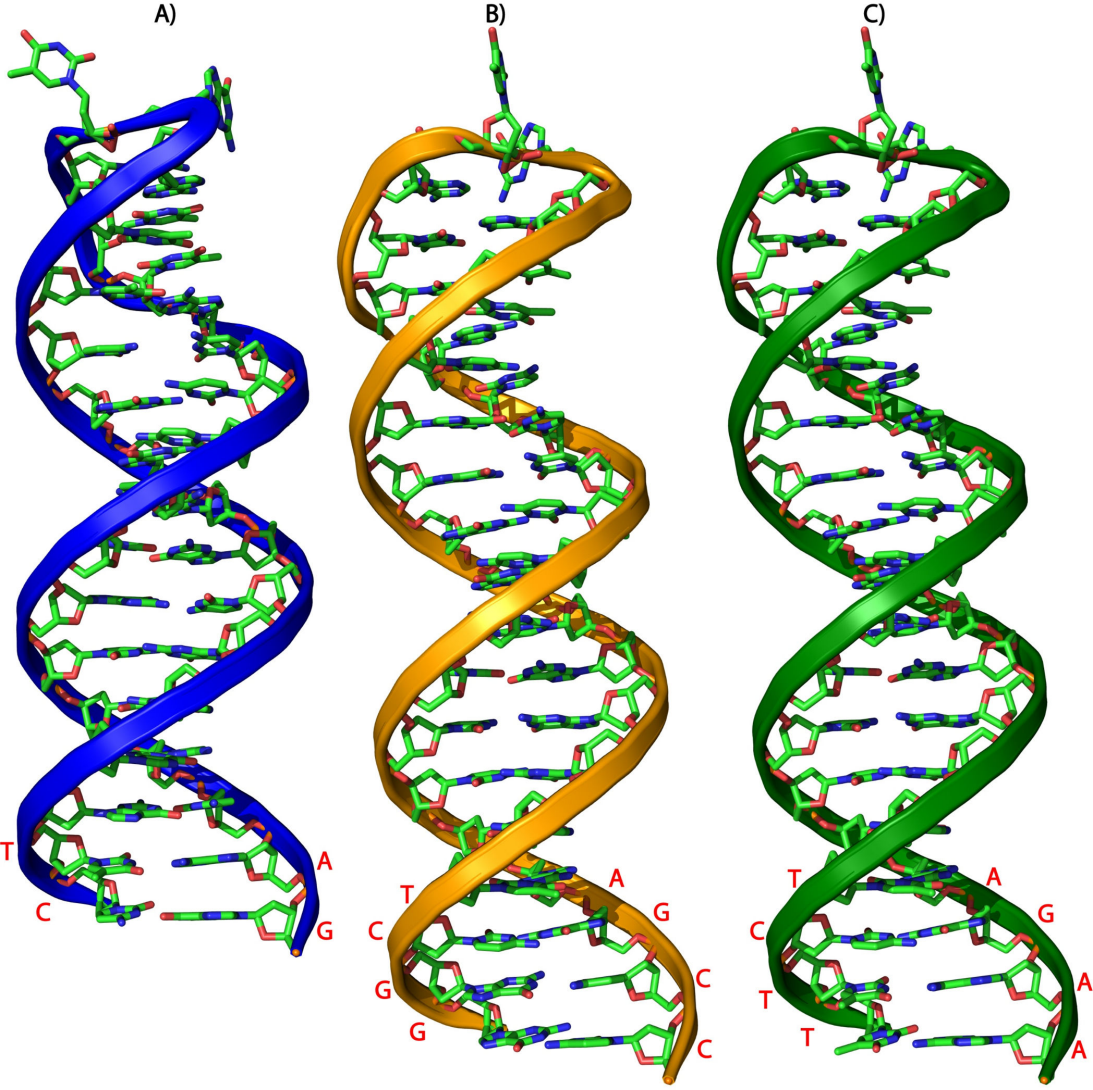
Molecular modeling of stem-loop segments. Wild type (A), MA1 (B) and MA2 (C). The structural organization of the stem-loop region (CR-SL) across all segments is presented using PyMOL’s schematic representation. This visualization highlights the overall architecture, shape, and spatial arrangement of each subunit within the stem-loop regions.

The molecular modeling of the stem-loop structure involved adding and mutating nucleotides in the neck region of the segments, namely WT (A) MA1 (B) and MA2 (C) (Fig. 3). The modeled stem-loop structures were refined and energy-minimized using University of California, San Francisco (UCSF) Chimera to eliminate steric clashes and unfavorable contacts.

### MD simulation

**Fig 4:**
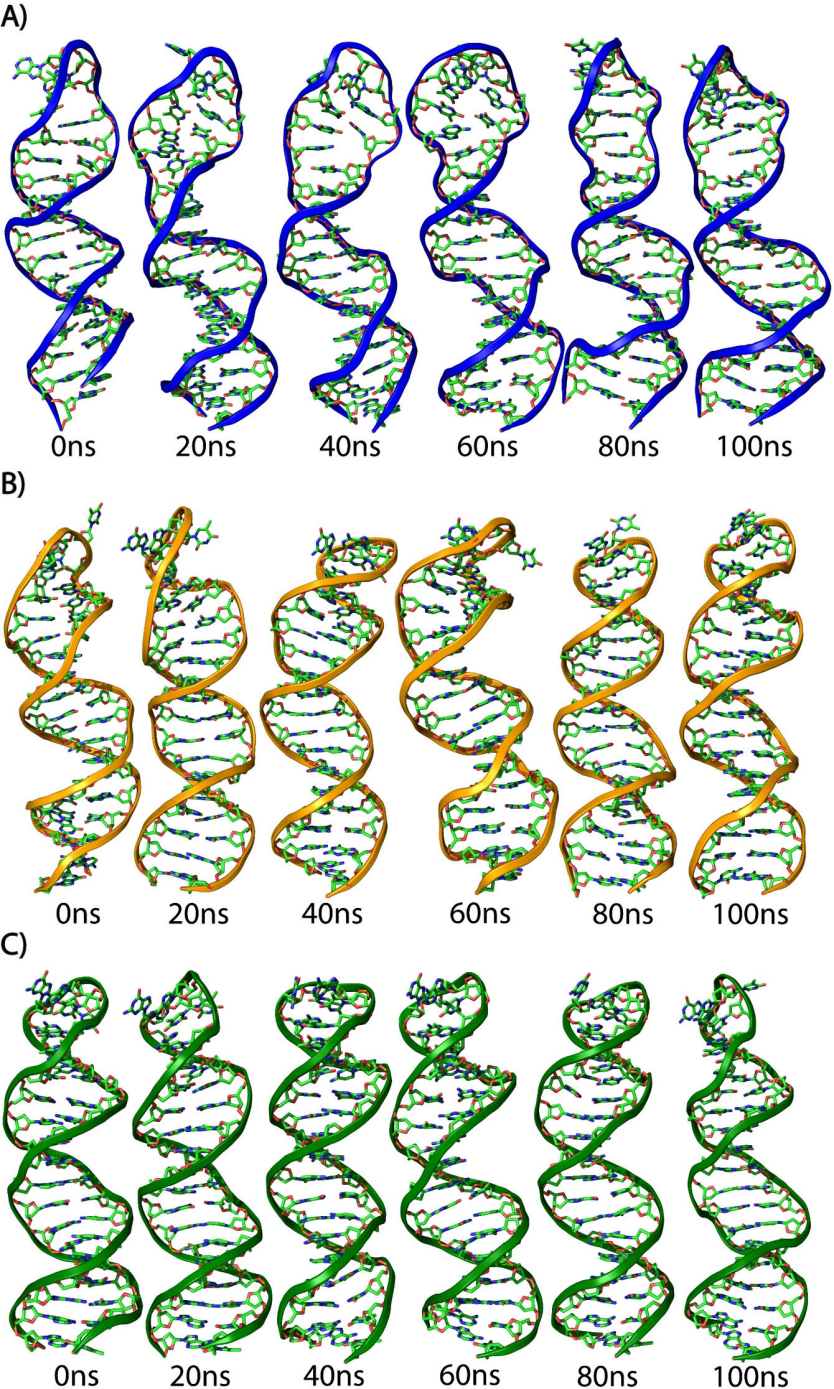
Snapshots of stem-loop structures from 0 to 100 ns for WT and mutants. MD simulations were performed to evaluate the structural stability and dynamic behavior of stem-loop structures for the WT and MA1 and MA2 over a 100 ns trajectory. All simulations were conducted in an explicit solvent at 300 K. Snapshots were extracted at intervals to observe changes in the structural conformation. (**A**) WT, (**B**) MA1, and (**C**) MA2.

**Fig 5:**
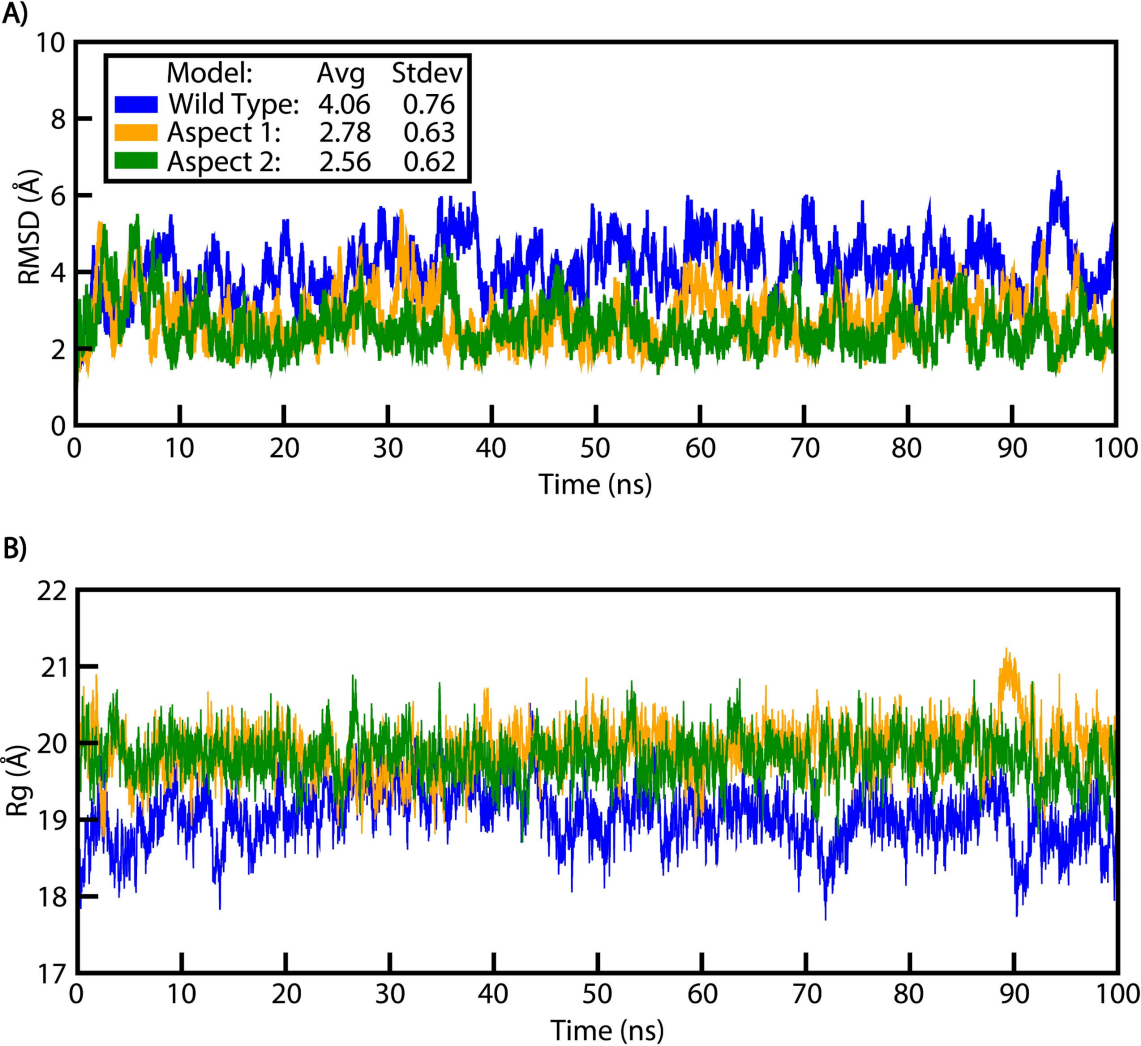
Post-simulation structural stability analysis of WT and mutants: (**A**) RMSD and (**B**) Rg analyses of WT, MA1, and MA2.

The structural stability, flexibility, and compactness of all systems were analyzed over a 100 ns trajectory to study the dynamic behavior of the WT and mutant stem-loop structures (Fig. 4). The topology calculations, including root-mean-square deviation (RMSD), radius of gyration (Rg), root-mean-square fluctuation (RMSF), and B-factor, were performed using explicit water simulations at 300 K. The mean RMSD values for the WT, MA1, and MA2 were 4.06 Å, 2.78 Å, and 2.56 Å, respectively (Fig. 5A). The higher mean RMSD of the WT structure likely reflects the inability of its first and last two nucleotides to properly bind, leading to destabilization. In contrast, the mutant aspects, with additional residues supporting the stem region, exhibited better overall stability, as evident in the simulation snapshots where the WT structure displayed a deformed loop and less stable overall structure (Fig. 4).

Structural compactness, as indicated by the Rg, correlated with stability. While the mutant aspects maintained a stable Rg around 20 Å throughout the 100 ns trajectory, the WT structure displayed lower Rg values (18–19 Å) due to its smaller size, resulting from fewer nucleotides (Fig. 5B). However, the fluctuations in the Rg plot for the WT indicate instability, consistent with the trend observed in the RMSD analysis.

**Fig 6:**
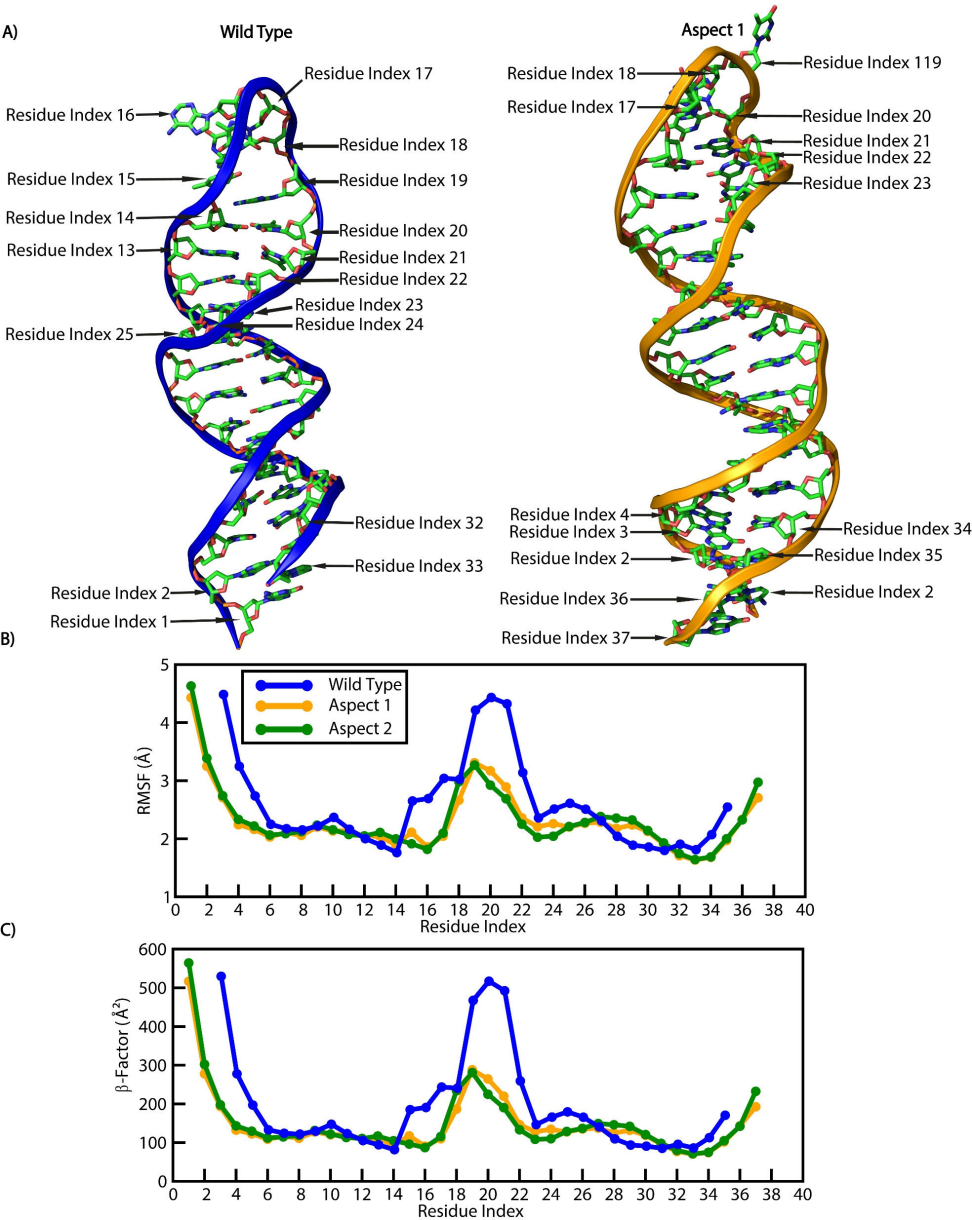
Conformational analysis of the WT and mutant aspects. (**A**) Overall structure and nucleotide location of WT and MA1. (B and C) RMSF and *B*-factor analysis of WT, MA1, and MA2 nucleotides. This indicated that the fluctuations of nucleotides of Aspects 1 and 2 systems of virus DNA R were less and more stable than the WT system throughout the simulation time.

Structural nucleotide fluctuations, as assessed by RMSF and B-factor plots, supported the above findings. Terminal residues of proteins and nucleotides typically exhibit higher RMSF and *B*-factor values. In this study, the WT loop region (nucleotides 12–22 Fig. 6A) showed significantly higher RMSF (>4 Å) and *B*-factor (>500 Å²) values compared to the mutant aspects having lower RMSF (<4 Å) and *B*-factor (<300 Å²; loop regions spanning nucleotides 14–24; Fig. 6A). The mutants exhibited reduced flexibility and improved stability due to stronger and more consistent nucleotide binding (Fig. 6B and C). The RMSF and *B*-factor peaks of the loop region of the WT were comparable to the terminal residue RMSF and *B*-factor values, highlighting its relative instability compared to the mutants.

### Free energy and per-residue binding energy analysis

**Fig 7:**
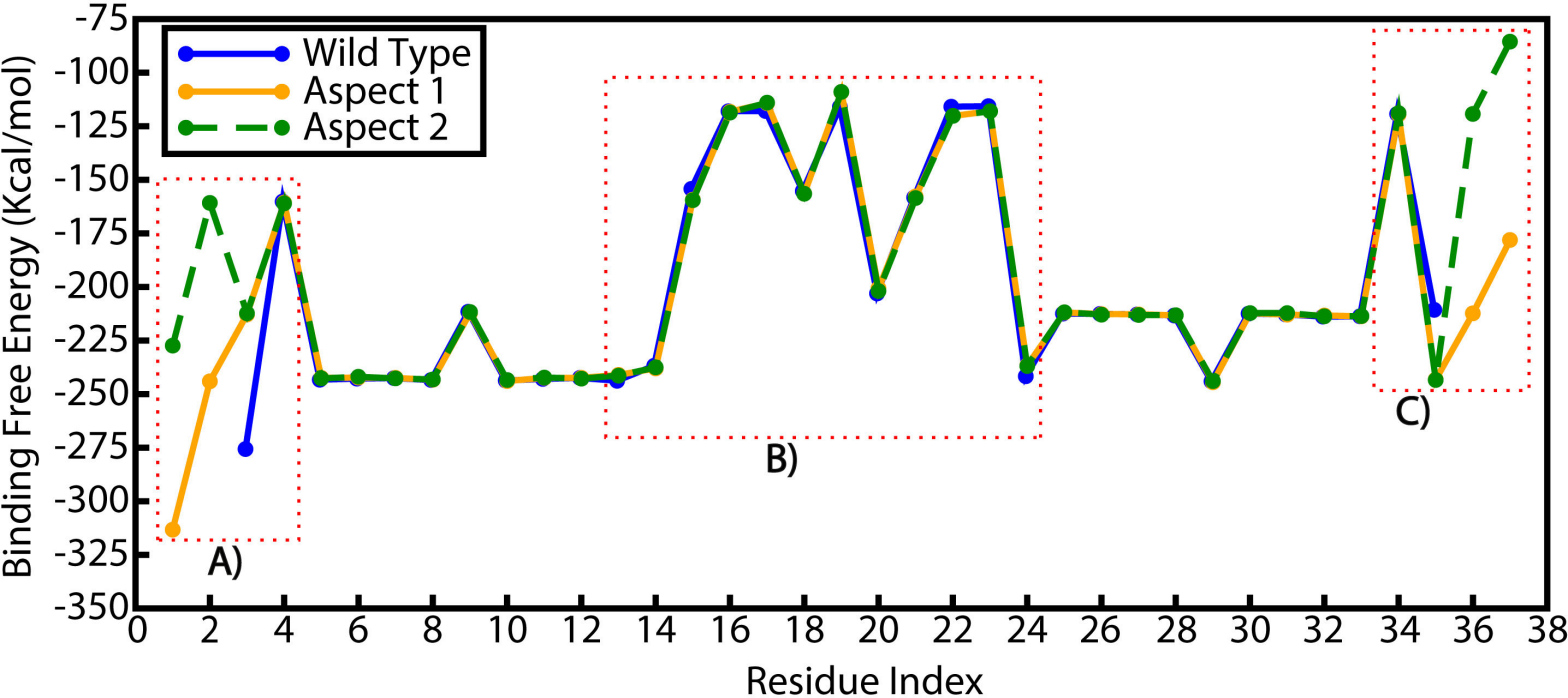
Residue energy calculations of WT, MA1, and MA2 and using molecular mechanics/generalized Born surface area analysis. The structural stability of the stem-loop structures was evaluated by calculating the binding free energy of individual residues. The analysis focused on (**A**) starting nucleotides (5′ terminus) of the stem region, (**B**) nucleotides in the loop or neck region, and (**C**) ending nucleotides (5′ terminus) of the stem region.

The stem-loop structure binding free energy indicated lower binding energies for MA1 and MA2 compared to the WT, indicating higher binding affinities (Fig. 7; Table S1). The starting terminal residue of MA2 had higher energy compared to those of MA1 and WT, which may be due to the residues consisting mostly of pyrimidines.

The per-residue decomposition graph provided insights into the energy contributions of individual nucleotides. The analysis revealed that nucleotides in the loop regions and terminal residues exhibited higher energy contributions (Fig. 7A through C). This is consistent with the inherent flexibility of terminal regions and the structural dynamics of the loop regions, which are more prone to higher energy fluctuations.

Conversely, the neck residues, located immediately adjacent to the termini, contributed to smaller energy variations and played a critical role in stabilizing the systems. These residues maintained base pair interactions and counteracted the destabilizing effects of the loop and terminal regions, ensuring the overall structural integrity of the stem-loop segments.

### Hydrogen bond analysis

Hydrogen bond formation was analyzed for all binding patterns. Bonds with distances less than 3 Å and angles greater than 135° were determined, and the terminal nucleotides of WT and mutant stem-loop structures were analyzed to assess their impact on system stability.

**Fig 8:**
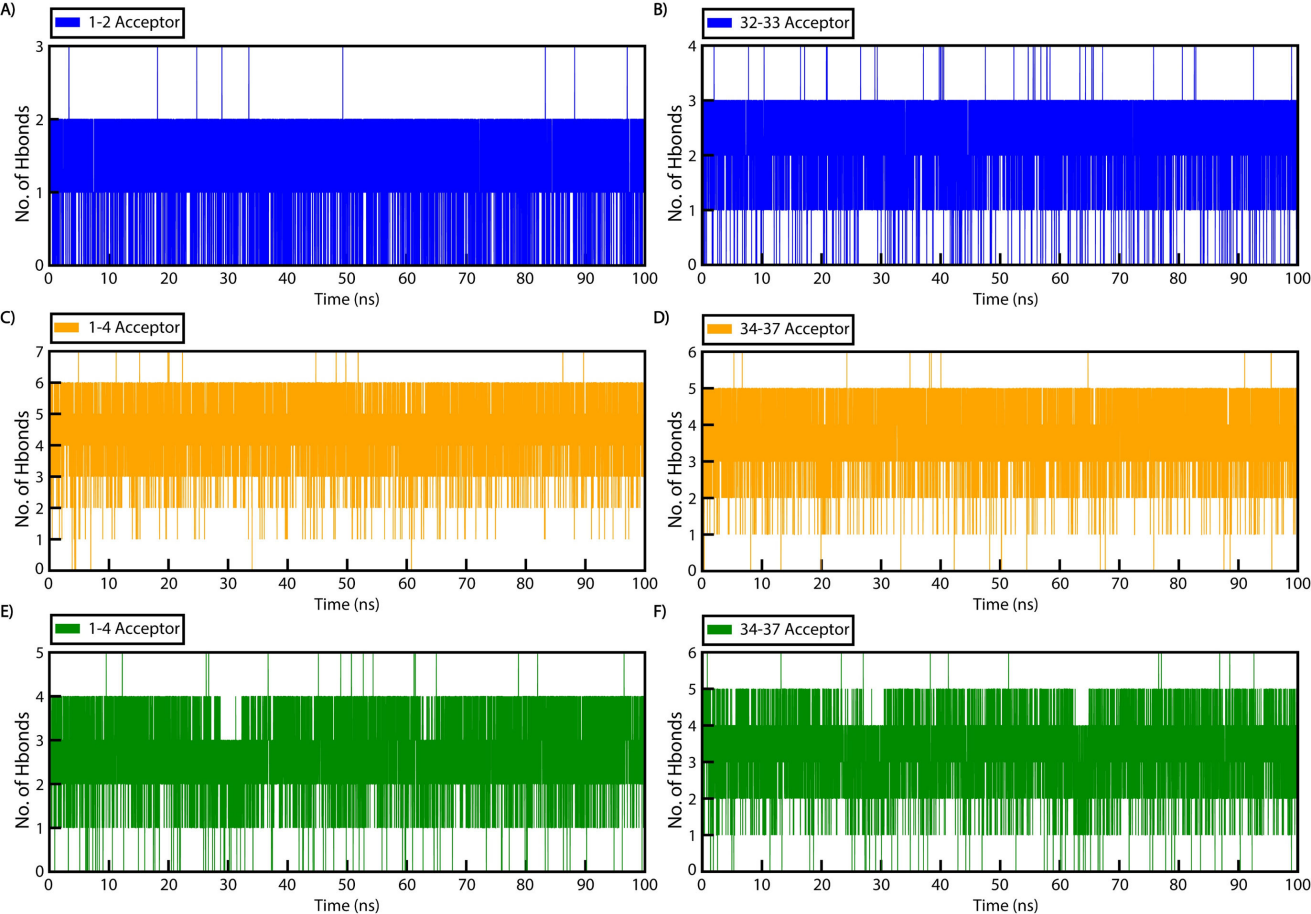
Hydrogen bond analysis of the WT and mutant aspects. Hydrogen bonding between the starting and ending nucleotides of the stem region in (A and B) WT, (C and D) MA1, and (E and F) MA2 during 100 ns simulation.

The total number of hydrogen bonds formed between the selected nucleotides (Fig. 8) and the residues involved, along with their occupancy or lifetime, was compared (Tables S2 to S4 and Fig. S1 to S3). In the WT, the number of hydrogen bonds with bond occupancy of more than 45% was five (Table S2 and Fig. S1 to S3), the most hydrogen bonds formed at a time during the simulation were seven (Fig. 8A and B), and the bonds formed among the terminal residues numbered 15 (Table S2). In contrast, for MA1 and MA2, the average numbers of hydrogen bonds with occupancy higher than 45% were 11 and 9 (Tables S3, S4 and Fig. S1 to S3), respectively; the maximum bonds formed at a time during simulation were 13 and 11, respectively (Fig. 8C through F); and the hydrogen bonds formed among the terminal nucleotides were 37 and 55 hydrogen bonds (Tables S3 and S4).

The results suggest that the mutant structures, with additional terminal residues and improved base pairing, were more stable than the WT due to the increased number and sustained occupancy of hydrogen bonds.

### Infectivity and quantitative PCR analysis of mutants in *Nicotiana benthamiana*

**Fig 9:**
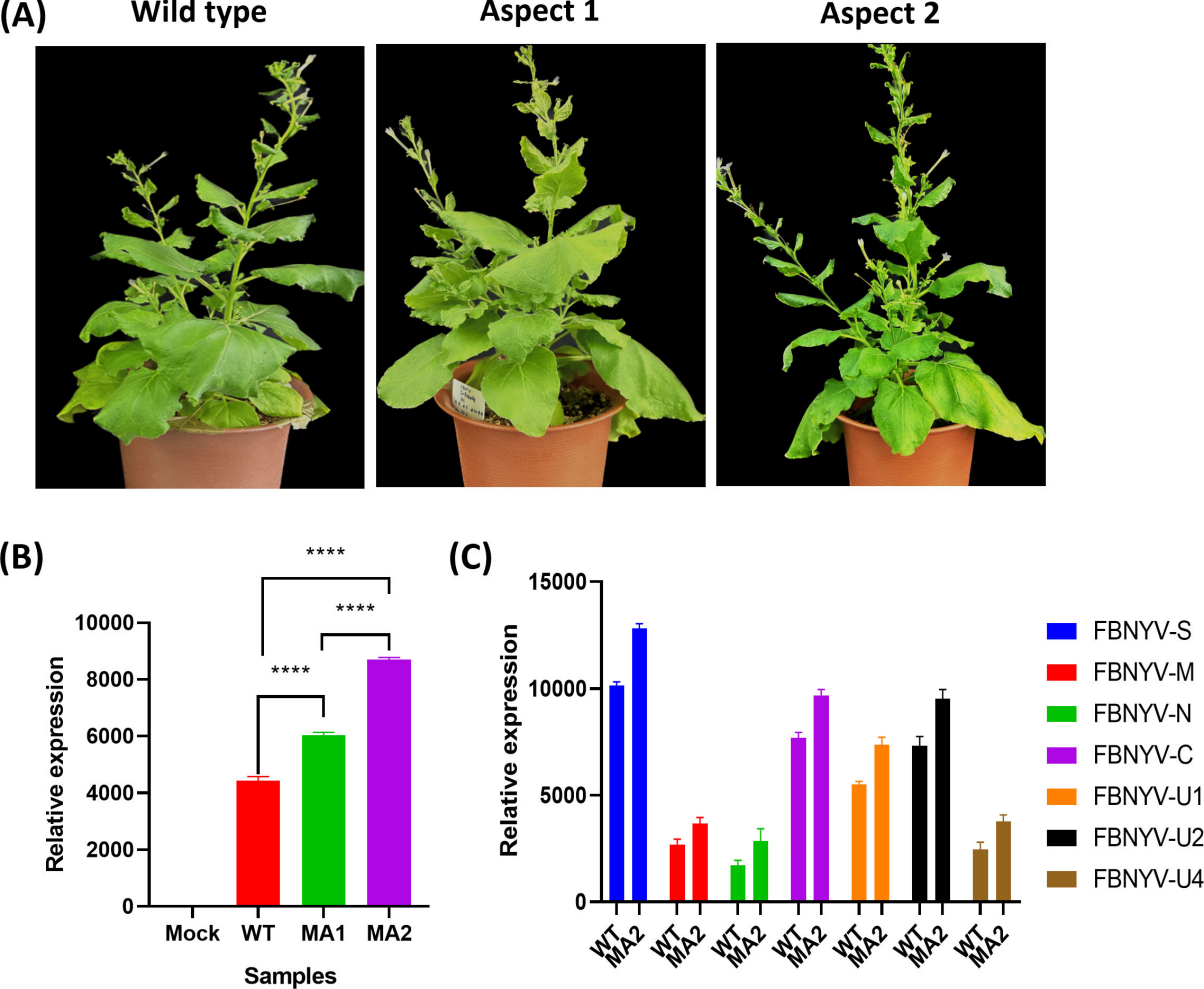
Infection development and symptoms analysis of WT, MA1, and MA2 in inoculated plants and their expression analysis through qPCR. (**A**) Symptoms analysis of WT, MA1, and MA2 in *N. benthamiana* at 28 dpi. Specifically, the symptoms that developed from FBNYV-DNA-R MA1 and MA2 were evaluated and compared with WT. (**B**) DNA-R of FBNYV and their expression in *N. benthamiana* through qPCR; asterisk represents the significant differences among three constructs expression determined by two-way ANOVA test. The *P*-values are ****P < 0.0001 for WT vs MA1 and ****P < 0.0001 for MA1 vs MA2 with 0.05 (95% CI). (**C**) qPCR analysis of expression of all segments in the infected *N. benthamiana*.

The agroinoculation of *N. benthamiana* plants with WT, MA1, and MA2 was conducted to evaluate the infectivity of the FBNYV-DNA-R mutants. Stunting and yellowing, two common FBNYV symptoms of infection, were observed in all WT, MA1, and MA2 inoculated plants, indicating effective systemic infection (Fig. 9A). Leaf tissue was collected at 28 days post-inoculation (dpi), and quantitative PCR (qPCR) was conducted with three replicates of each construct to confirm the replication of the virus by using specific primers that target the viral genome (Table S5).

The infectivity of MA1 and MA2 varied. While MA1 exhibited slightly stronger and earlier symptom onset than the WT, MA2 agroinoculation resulted in more severe symptoms than the WT and MA1 (Fig. 9A). MA1 had a delayed onset of symptoms relative to MA2, with some plants presenting either lesser or no observable symptoms by 28 dpi. Additionally, qPCR data showed that plants infected with MA1 and MA2 exhibited viral DNA accumulation 25% and 51% higher than that of the WT, respectively. The expression of all segments of FBNYV in plants infected with mutants was slightly elevated relative to the WT (Fig. 9C).

## DISCUSSION

Nanoviruses are multipartite, single-stranded DNA viruses characterized by a unique genomic architecture in which each segment is independently encapsulated. Each segment encodes a distinct protein with a specific function, as mentioned in the introduction. The IR in the non-coding region plays a crucial role in viral replication, as it contains essential motifs that regulate functional activity. The IR includes iterons, short repeated sequences that serve as binding sites for replication-associated proteins. Additionally, the CR-SL structure within the inverted repeat features a nonanucleotide (TAGTATTAC) motif, which serves as the nicking site for replication initiation (21, 22). In RCR-dependent viruses, such as nanoviruses and geminiviruses, this region is the cleavage site for the Rep protein, marking the initiation of replication (2, 23).

Previous studies on geminiviruses demonstrated that mutations in the stem-loop region affect viral DNA accumulation and infectivity. For example, in *Tomato yellow leaf curl virus* and Maize streak virus, alterations in the loop and nonanucleotide sequence impacted Rep recruitment and cleavage efficiency, leading to variations in replication kinetics and symptom expression (24, 25). Similarly, in Beet curly top virus and Wheat dwarf virus, modifications in the hairpin structure influenced viral strand synthesis and symptom severity (26, 27). These studies emphasized the regulatory importance of the stem-loop structure in replication initiation and viral accumulation.

To better understand these mechanisms in nanoviruses, our approach integrated computational modeling and experimental validation through agroinoculation and qPCR analysis. Prior studies, particularly on MDV, focused on sequence alignment, motif analysis, and structural characterization of stem-loop variations, leading to the hypothesis that both stem-loop length and base-pairing composition influence segment expression levels (28). Similar to these studies, we constructed mutants based on both stem-loop length and composition, modifying the neck region to either extend or reduce base pair interactions. However, a key motivation of our study was to determine whether these regulatory behaviors are conserved across different nanoviruses or vary depending on the virus and host system. Our methodology differed from that of previous studies in terms of the structural modeling approach, simulation conditions, and the integration of computational and experimental analyses, allowing a direct assessment of how stem-loop modifications affect replication efficiency.

Notably, stem-loop variation in FBNYV was observed exclusively in the FBNYV-U1 and FBNYV-C segments, which contain shorter neck regions (nine nucleotides) than other segments (11 nucleotides). In-silico analyses predicted that stem-loop mutants with additional nucleotides in the stem region would be structurally more stable due to improved base pairing compared to the WT. Studies have shown that hairpin structure stability depends on stem length and sequence composition, where the stem region exhibits lower RMSD values than the loop, contributing less to high-energy fluctuations in the system (29). Our MD simulations confirmed that modifications in MA1 and MA2 increased structural stability, reflected in the lower RMSD values and greater hydrogen bonding relative to the WT structure. These findings align with computational studies showing that GC-rich stems enhance thermodynamic stability (29).

**Fig 10:**
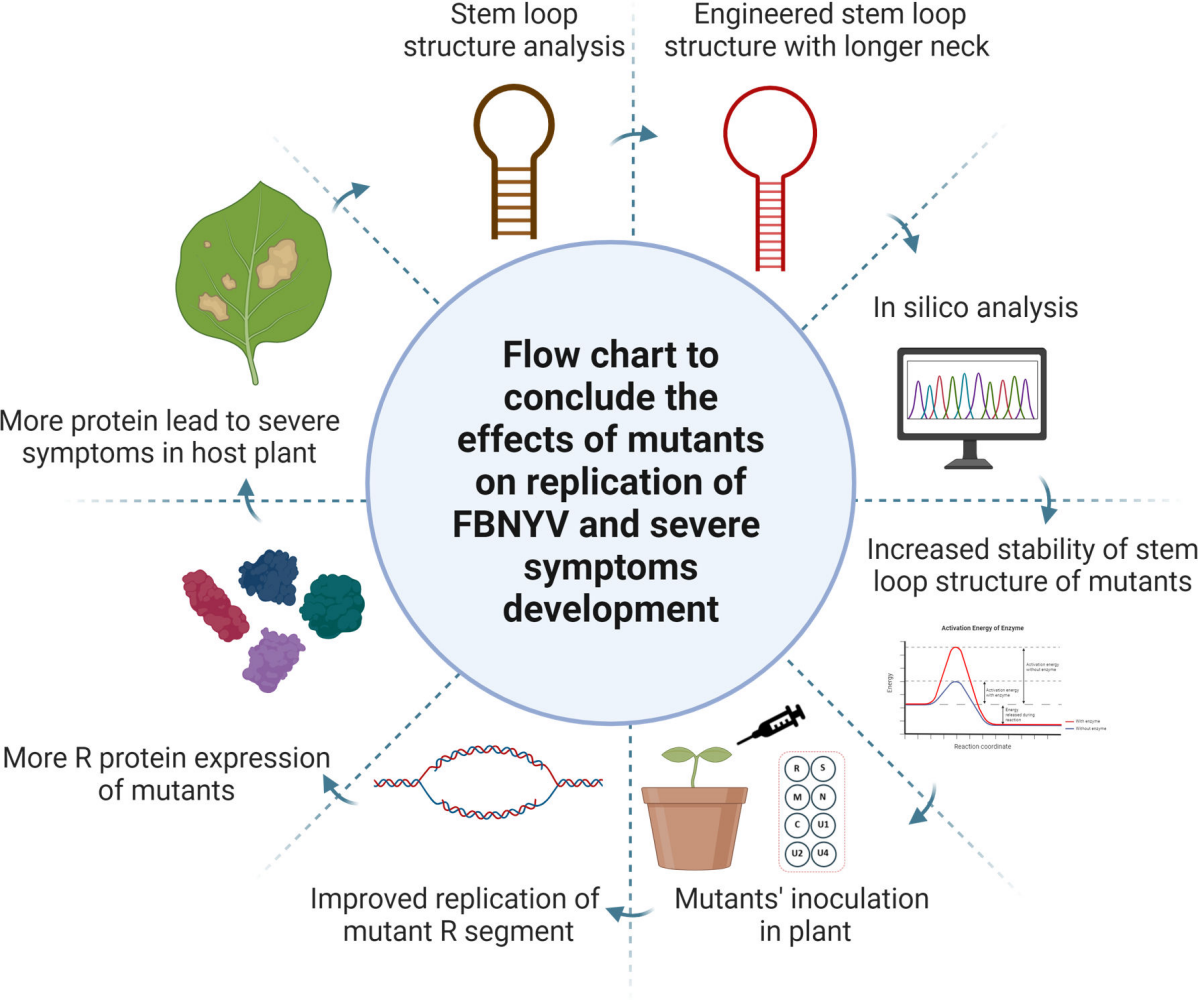
Effects of mutants on replication of FBNYV and severe symptoms development. The figure shows the addition of nucleotides in the neck region to create mutants’ aspects. The mutants show an increase in stem-loop stability as compared to WT stem-loop. After inoculation in the host plant, the mutant stem-loop caused an increase in the replication of FBNYV-R segment, which led to an increase in replication protein and, as a result, more replication of other segments. Increased replication of all segments of FBNYV caused severe symptoms development in the host plant as compared to WT infection.

qPCR-based expression analysis revealed that stem-loop modifications influenced viral replication efficiency in nanoviruses. Infection assays in *N. benthamiana* demonstrated that plants infected with MA1 and MA2 mutants accumulated higher viral DNA than those infected with WT FBNYV, supporting that enhanced stem-loop stability promotes replication efficiency. This is consistent with observations in RCR-dependent viruses, in which stable secondary structures correlated with increased replication efficiency (24, 25). Interestingly, symptom severity varied among mutants, suggesting additional regulatory roles for the stem-loop structure beyond replication (Fig. 10). Differences between MA1 and MA2 indicate that stem-loop modifications may also impact systemic movement or host-virus interactions. This aligns with broader findings that viral regulatory elements influence multiple aspects of the infection cycle beyond replication alone.

Our study revealed that extending the stem-loop neck region increased replication efficiency, with MA1 and MA2 exhibiting higher viral accumulation than the WT. This agrees with previous findings that longer stem-loops (11 nucleotide pairings) in MDV segments result in higher expression levels than shorter variants (nine nucleotide pairings) (28). Furthermore, we found that base-pair composition significantly influenced replication efficiency, consistent with studies showing that G-C pairing at position 7 enhances expression compared to T-A pairing (28). Although FBNYV and MDV differ in genome composition and host range, we observed a similar trend, suggesting that stem-loop-mediated replication regulation may be a conserved mechanism across nanoviruses, independent of host-virus interactions.

The results also align with findings in geminiviruses, indicating that stable secondary structures optimize Rep binding and cleavage, facilitating efficient rolling-circle replication (23). However, unlike geminiviruses, where drastic modifications in the stem-loop region often impair replication (24), our study demonstrated that moderate modifications (e.g., increasing stem length by a few base pairs) enhance replication rather than disrupt it. This suggests that nanoviruses may tolerate a greater degree of structural variation while maintaining functional efficiency, likely due to their multipartite genome structure, which provides greater segment regulation flexibility.

While our study highlighted conserved regulatory mechanisms, the responses of nanoviruses to stem-loop variations may be species specific. Although the trend of longer stem-loop enhancing replication efficiency appears consistent, the precise effects of structural modifications may vary depending on host factors, viral genome organization, or segment compatibility within the multipartite system. Future studies should investigate whether similar regulatory effects occur across additional nanoviruses in different host species. Exploring the interplay between stem-loop stability, Rep binding, and host factors will further elucidate the molecular mechanisms governing nanovirus replication. These insights could have broader implications for developing antiviral strategies targeting conserved regulatory elements in multipartite viruses.

## MATERIALS AND METHODS

### Virus source and infection test

**Fig 11:**
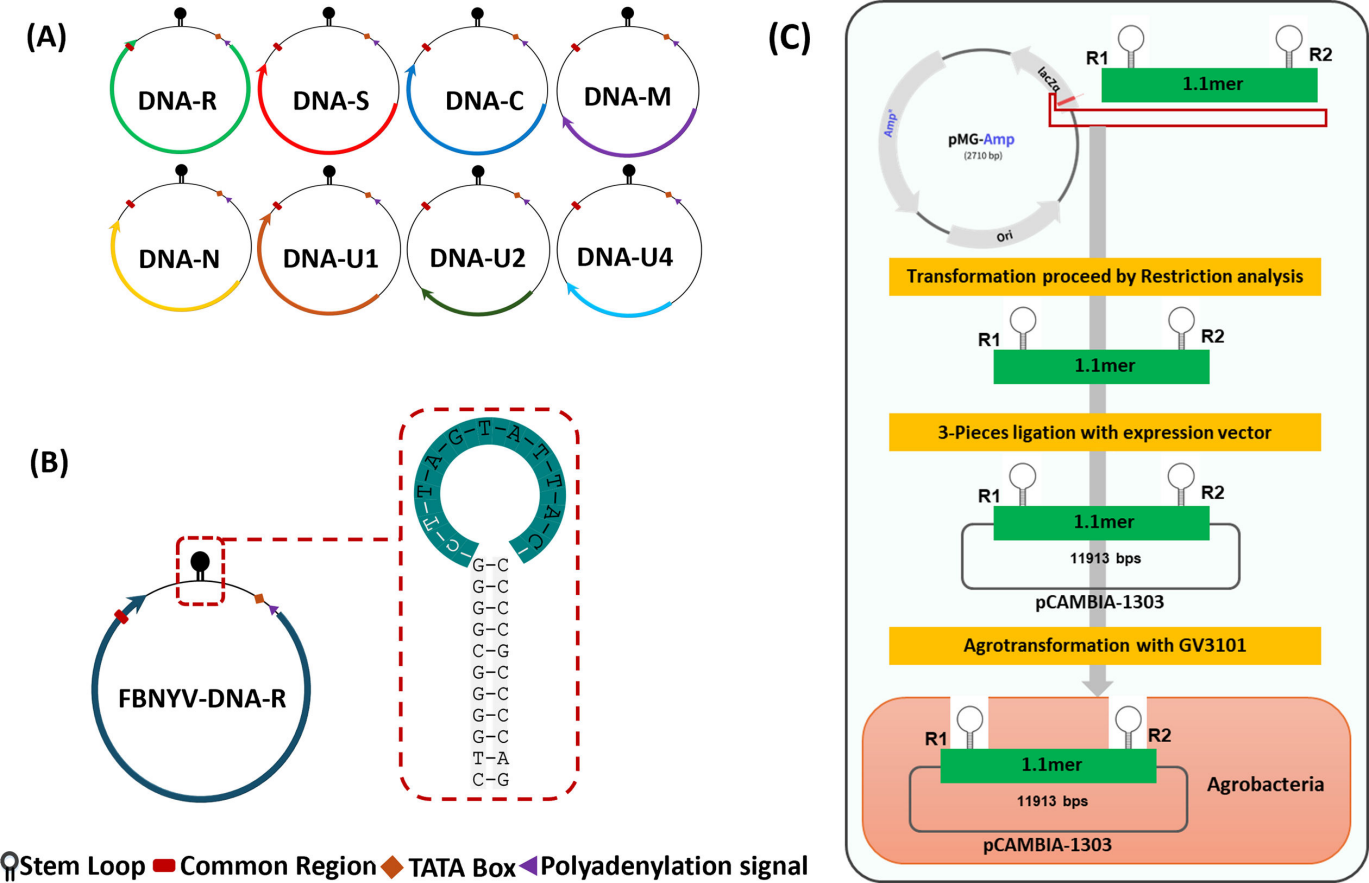
Genomic structures of nanovirus with eight segments along with the stem-loop structure and sequence and strategies to construct infectious clone. (**A**) Nanovirus genome consisting of eight independently encapsidated DNA segments. DNA-R produces master rep protein, DNA-S coat protein, and DNA-C, DNA-M, and DNA-N produce Clink, movement, and nuclear shuttle protein, respectively. (**B**) DNA-R genomic component of FBNYV with stem-loop structure and nonanucleotide sequence. (**C**) Flow chart of the construction of synthesized clones into expression vectors for inoculation in plants.

As FBNYV has not been reported in Korea, we could not construct the infectious clone in our lab. FBNYV isolate *EV1-93* (30) was artificially synthesized by Macrogen (Korea; https://dna.macrogen.com). Genomes of the FBNYV DNA segments (DNA-C, DNA-R, DNA-M, DNA-N, DNA-S, DNA-U1, DNA-U2, and DNA-U4; Fig. 11A) were cloned to check their infectivity and impact variance in the host plant (data not shown). Two partial genome fragments were amplified for each virus sequence, and infectious clones were generated using primer sets. The clones were ligated into cloning vector pGEM-T Easy, followed by sequencing and restriction digestion. The clones were then transformed into *Escherichia coli* strain DH5α and *Agrobacterium tumefaciens* strain GV3101 (Fig. 11B). The transformed and untransformed GV3101 *Agrobacterium* strains were cultured in Luria-Bertani broth containing strain-specific antibiotics. Agroinoculation was performed on *N. benthamiana* plants, with mock inoculations performed using GV3101 as a control. At 28 dpi, the infection was confirmed, and segments were detected using PCR.

### FBNYV sequence analysis and alignment

Sequences from all segments of FBNYV were obtained from GenBank (http://www.ncbi.nlm.nih.gov). The sequences were aligned at a common position, specifically with the nonanucleotide (5ʹ-TAATATT//AC-3ʹ) at the nicking site. Using QIAGEN CLC sequencer view 8.0 (https://digitalinsights.qiagen.com/), ORFs and IRs were separated and aligned with the corresponding ORFs and IRs from other segments (Fig. 1A).

### In-silico mutant sequence retrieval based on different aspects and molecular modeling

Mutant sequences were retrieved and analyzed based on several criteria, including nucleotide addition in the stem-loop neck region with different variations (Fig. 2). Computational tools and databases, including GenBank and Multalin (http://multalin.toulouse.inra.fr/multalin/), were used to identify and align mutant sequences for further comparative analysis. The structure of the stem-loop was modeled using WinCoot (31). Nucleotides were added and mutated in modeled structures, followed by structure minimization using University of California, San Francisco (UCSF) Chimera (32) to eliminate bad contacts and resolve steric clashes in the structure.

### Molecular modeling of aspects of stem-loop sequence

The molecular modeling of the stem-loop structure involved adding and mutating nucleotides in the neck region of the segments, namely WT (A) MA1 (B) and MA2 (C) (Fig. 3). The modeled stem-loop structures were refined and energy-minimized using University of California, San Francisco (UCSF) Chimera to eliminate steric clashes and unfavorable contacts.

### MD simulations

The structural dynamics of the stem-loop structures were assessed utilizing AMBER 22 (33). System configurations were created with AMBER’s DNA OL15 force field, supplemented by a terminal monophosphate library for the terminal residues (20). The complexes were solubilized in a 9 Å water box containing OPC water molecules (34) applying periodic boundary conditions. To neutralize system charges and mimic physiological conditions, counterions (Na^+^ and Cl^−^) were added, maintaining an ionic strength of 0.150 mM (35). The systems were subjected to a two-step energy minimization to eliminate bad contacts and steric clashes by first minimizing only the water molecules and then minimizing both water molecules and the stem-loop structures. Subsequently, the systems were gradually heated from 0 to 300 K under an constant Number of particles, Volume, and Temperature (NVT) ensemble over 25 ps, with a timestep of 0.5 fs, while restraining the stem-loop structure. This was followed by a four-step equilibration process using the constant Number of particles, Pressure, and Temperature (NPT) ensemble at a constant temperature of 300 K and 1 atm pressure. Each step gradually reduced the restraints on the stem-loop structures, followed by the last step of the restraint-free equilibration phase, lasting two ns, with a timestep of 0.5 fs.

MD simulations were conducted for 100 ns with a timestep of 1 fs, saving coordinates every 10 ps for analysis. Post-simulation analyses included RMSD, Rg, B-factor, and RMSF of the sugar-phosphate backbone and were performed using the CPPTRAJ module of AmberTools23 and visualized in xmgrace (36). These analyses provided insights into the stability, flexibility, and conformational dynamics of the stem-loop structures.

### Energy calculation

The free energy of the stem-loop structure and per-residues were calculated using the molecular mechanics/generalized Born surface area and Poisson Boltzmann surface area algorithms using AMBER22 (33, 37). Each MD trajectory was utilized to provide 2,000 snapshots of simulation per trajectory to calculate the binding free energy and energy decomposition. The whole system energy and binding free energy of each nucleotide were calculated by combining all the interactions with all other nucleotides in the system.

### Hydrogen bond analysis

Hydrogen bonding analysis for all three stem-loop structures was conducted using the CPPTRAJ module (38). The total number of hydrogen bonds and their lifetime occupancy were evaluated for each system. Specifically, analyses focused on the first and last four nucleotides of both the mutated stem-loop structures and the first and last two residues of the WT structure to assess hydrogen bond patterns and their impact on segment stability. The hydrogen bond graphs were visualized using xmgrace (36) and GNUPLOT (39) for a clear representation of binding patterns.

### Mutant construction

Two mutant versions of the FBNYV-DNA-R segment were designed (Fig. 2) and constructed based on neck region variation in stem-loop using the Q5 Site-Directed Mutagenesis Kit (NEB, MA, USA; Fig. 2B): (i) 13 nucleotide length neck regions with G-C pairing addition (MA1) and (ii) 13 nucleotide length neck regions with T-A pairing variation (MA2). For both mutants, four nucleotides were added at the end of the stem-loop neck region of DNA-R. Infectious clones of each mutant were constructed in the same way as described earlier in the WT case. Each virus was inoculated in nine *N. benthamiana* plants, along with control plant inoculation with GV3101 *Agrobacterium*.

### Nucleic acid extraction

To determine the infectivity, leaf tissue samples of FBNYV agroinoculated plants (WT, MA1, and MA2) were collected at 14 and 28 dpi. All samples were stored at −20°C until processing. All samples were sterilized with 70% ethanol for 20–30 s and left to dry from the airflow under the fume hood. Total DNA was extracted from leaf tissue samples using the Viral Gene-Spin Viral DNA/RNA Extraction Kit (iNtRON Biotechnology) following the manufacturer’s instructions for further analysis.

### PCR and qPCR analysis

Using the leaf tissue samples, PCR was conducted to assess infectivity using primers designed to amplify each DNA segment of FBNYV. Expression levels of both original and mutant segments were evaluated via qPCR. The experiment was conducted with specific primer sets (Table S5) and the SYBR Premix Ex Taq (Tli RNase H Plus, TaKaRa, Shiga, Japan). The Rotor-Gene Q thermocycler (QIAGEN, Hilden, Germany) was employed to perform the PCR cycling process, which consisted of an initial denaturation at 95°C for 5 min, followed by 40 cycles of denaturation at 95°C for 10 seconds, annealing at 58°C for 15 seconds, and extension at 72°C for 20 seconds. The annealing temperature was adjusted in accordance with the melting temperature of a particular primer, and each reaction was conducted at least three times. The 2^−ΔΔCt^ method was employed to undertake data analyses (40). Statistical analyses were performed using the *t*-test in the GraphPad Prism software (GraphPad Software, Boston, MA, USA).

## SUPPLEMENTAL MATERIAL

Supplemental material spectrum.00941-25-s0001.pdf Figures S1 to S4; Tables S1 to S5.

ASM does not own the copyrights to Supplemental Material that may be linked to, or accessed through, an article. The authors have granted ASM a non-exclusive, world-wide license to publish the Supplemental Material files. Please contact the corresponding author directly for reuse.
