# Supplementary material for: Regulatory role of stem-loop structures in Faba bean necrotic yellows virus replication efficiency
Source: Microbiol Spectr. 2025 May 27;13(7):e00941-25. doi: 10.1128/spectrum.00941-25 (PMC12211011; doi:10.1128/spectrum.00941-25)
Supplement: Supplemental material — Figures S1 to S4; Tables S1 to S5. [file spectrum.00941-25-s0001.pdf]

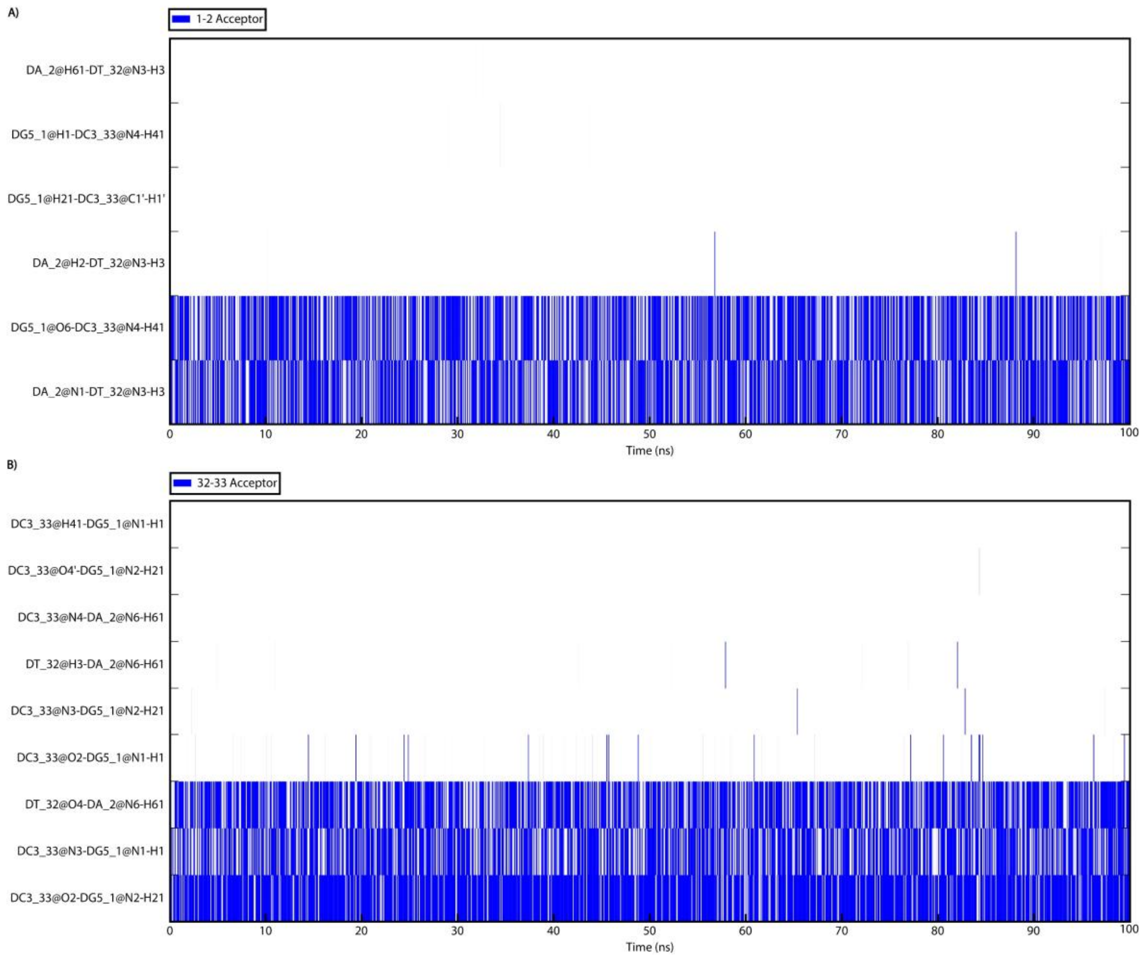

Supplementary Fig. S1. Hydrogen bond occupancy of the wild type stem-loop structure with (A) the first 2 nucleotides as hydrogen acceptor and (B) the last 2 nucleotides as hydrogen acceptor.

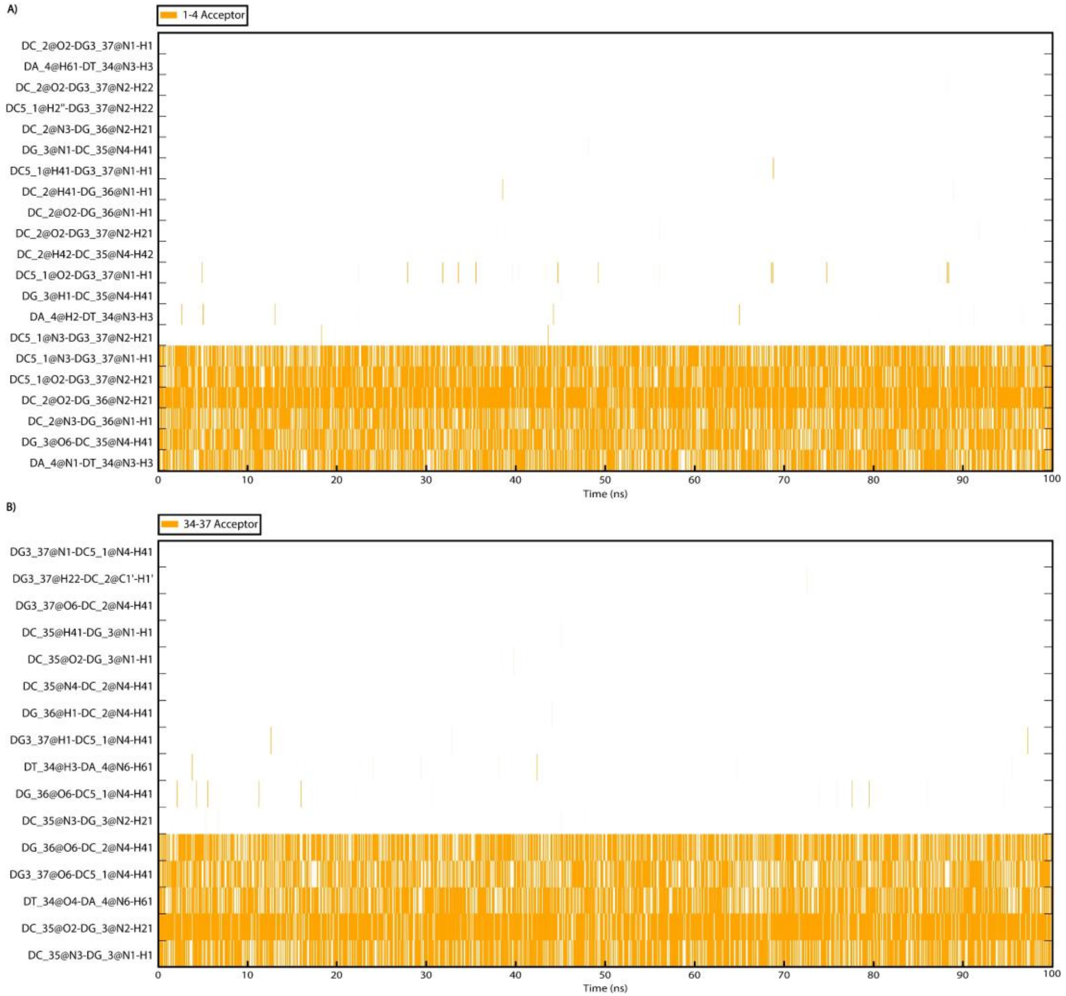

Supplementary Fig. S2. Hydrogen bond occupancy of the mutant aspect 1 stem-loop structure with (A) the first 4 nucleotides as hydrogen acceptor and (B) the last 4 nucleotides as hydrogen acceptor.

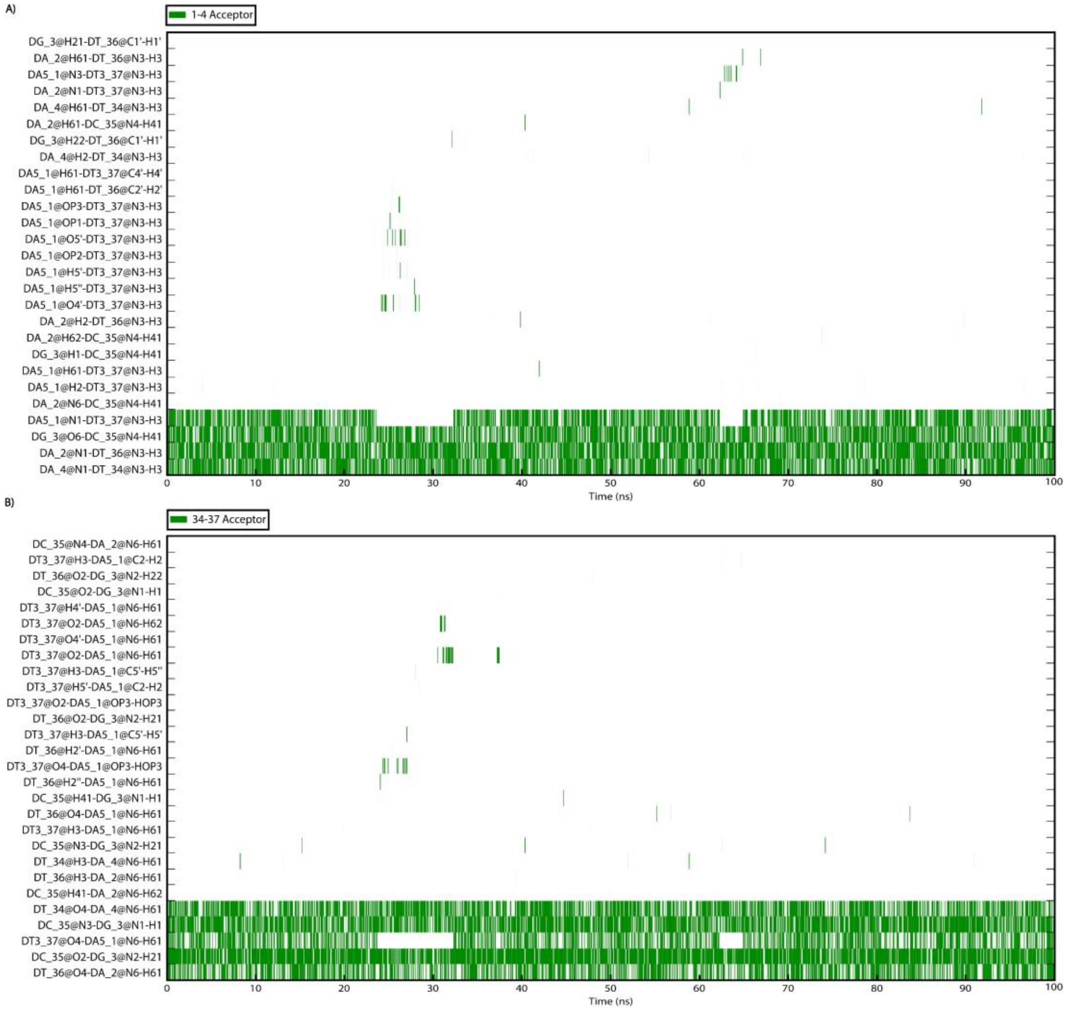

Supplementary Fig. S3. Hydrogen bond occupancy of the mutant aspect 2 stem-loop structure with (A) the first 4 nucleotides as hydrogen acceptor and (B) the last 4 nucleotides as hydrogen acceptor.

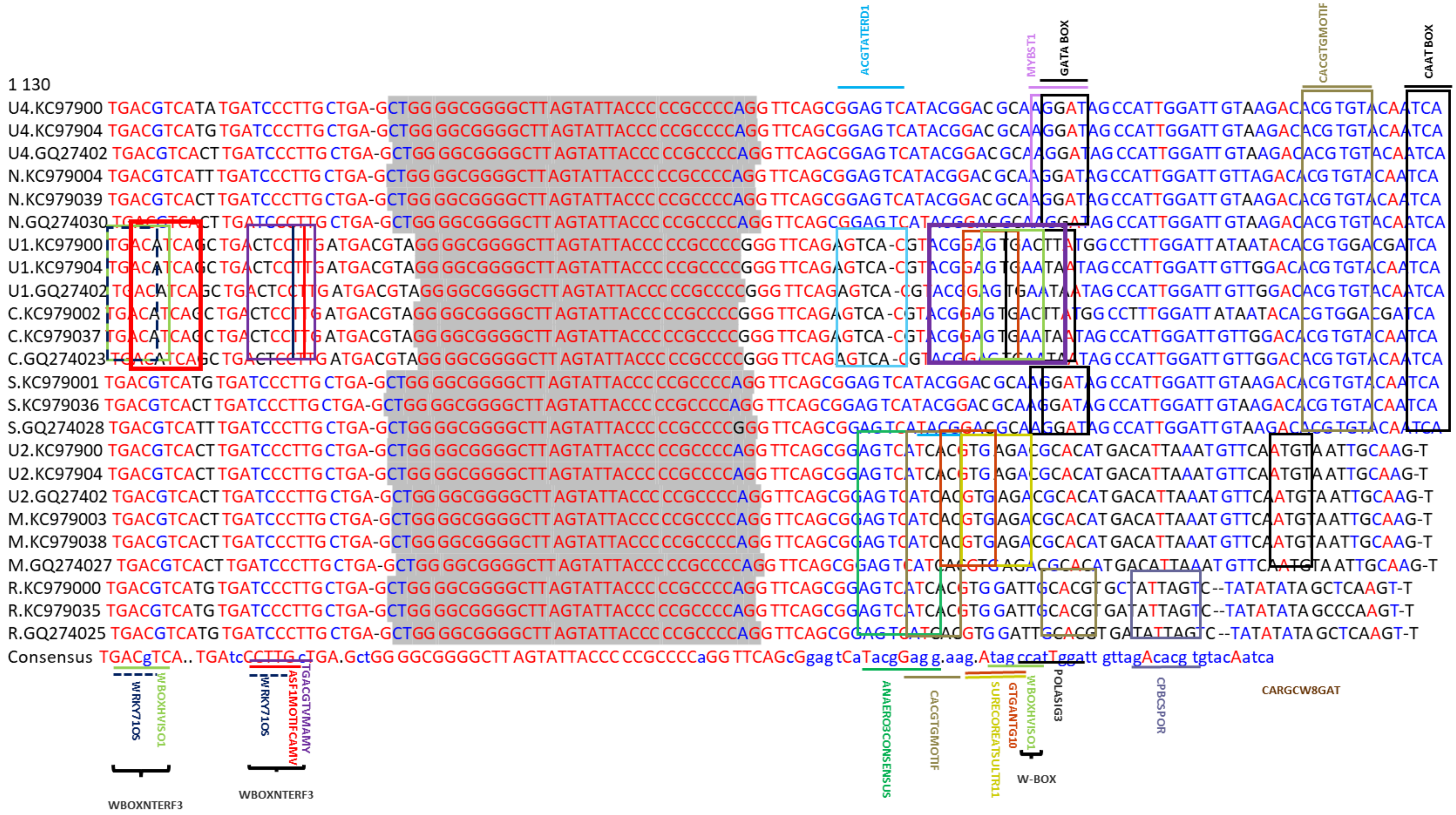

Supplementary Fig. S4. : Analysis of Stem loop sequence in the SL-CR of all segments of FBNYV. Among FBNYV segments, DNAs C and U1 possess the same composition of motifs in the SL-CR. But different in sequence with other segments.

Supplementary Table S1. Free energy and Per-Residue Binding Energy. As the values indicated that lower binding energies which translates to higher binding affinities for MA1 and MA2 compared to the wild type.

POISSON BOLTZMANN							
**Model Name**	**vdW**	**EEL**	**EPB**	**ENPOLAR**	ΔG_{gas}	ΔG_{solv}	ΔG_{total}
**Wild Type**	**-570**	**7448**	**-11329**	**664**	**4430**	**-11271**	**-6841**
**Aspect 1**	**-647**	**9353**	**-13589**	**741**	**5753**	**-13521**	**-7767**
**Aspect 2**	**-651**	**9034**	**-13610**	**742**	**6125**	**-13536**	**-7411**
GENERALIZED BORN							
**Model Name**	**vdW**	**EEL**	**EGB**	**ESURF**	ΔG_{gas}	ΔG_{solv}	ΔG_{total}
**Wild Type**	**-570**	**7448**	**-11080**	**43**	**4430**	**-11036**	**-6605**
**Aspect 1**	**-647**	**9353**	**-13317**	**48**	**5753**	**-13269**	**-7516**
**Aspect 2**	**-651**	**9034**	**-13334**	**48**	**6125**	**-13286**	**-7161**

Supplementary Table S2. Hydrogen bond analysis of wild type stem loop structure. The analysis determined the total number of maximum hydrogen bonds formed between the selected nucleotides. Also, the residues involved in the hydrogen bonds observed.

**1-2 Acceptor**						
**DonorResidue**	**DonorAtom**	**AcceptorResidue**	**AcceptorAtom**	**AvgDist**	**AvgAng**	**Occupancy**
DT32	N3	DA2	N1	2.8992	164.5013	0.6677
DC33	N4	DG1	O6	2.8679	162.9842	0.6463
DT32	N3	DA2	H2	2.9384	138.7187	0.0014
DC33	N4	DG1	H1	2.8201	136.1287	0.0007
DC33	C1'	DG1	H21	2.9764	145.4761	0.0002
DT32	N3	DA2	H61	2.9324	137.1660	0.0001
**32-33 Acceptor**						
**DonorResidue**	**DonorAtom**	**AcceptorResidue**	**AcceptorAtom**	**AvgDist**	**AvgAng**	**Occupancy**
DG1	N2	DC33	O2	2.8341	162.1242	0.8557
DA2	N6	DT32	O4	2.8591	163.6551	0.7070
DG1	N1	DC33	N3	2.9131	163.5541	0.6620
DG1	N1	DC33	O2	2.8990	146.7287	0.0184
DA2	N6	DT32	H3	2.8439	137.6102	0.0012
DG1	N2	DC33	N3	2.9489	151.1516	0.0009
DG1	N2	DC33	O4'	2.9431	144.2321	0.0003
DG1	N1	DC33	H41	2.7698	141.1477	0.0001
DA2	N6	DC33	N4	2.8471	138.3656	0.0001

Supplementary Table S3. Hydrogen bond analysis of mutant aspect 1. The values showed that all different hydrogen bonds formed among the terminal nucleotides of the MA1 were 37 and the average numbers of hydrogen bonds having occupancy higher than 45 % were 11.

1-4 Acceptor						
DonorResidue	DonorAtom	AcceptorResidue	AcceptorAtom	AvgDist	AvgAng	Occupancy
DG36	N2	DC2	O2	2.8336	163.7406	0.8809
DG37	N2	DC1	O2	2.8465	160.4642	0.7695
DG36	N1	DC2	N3	2.9120	165.8892	0.7370
DC35	N4	DG3	O6	2.8649	163.9761	0.7354
DG37	N1	DC1	N3	2.9096	163.5472	0.7023
DT34	N3	DA4	N1	2.8981	164.9419	0.6807
DG37	N1	DC1	O2	2.9087	147.3657	0.0053
DT34	N3	DA4	H2	2.9153	141.6890	0.0020
DG37	N2	DC1	N3	2.9426	149.7090	0.0014
DC35	N4	DG3	H1	2.7485	138.0542	0.0009
DG37	N2	DC2	O2	2.9084	143.4738	0.0008
DG37	N1	DC1	H41	2.7919	138.6263	0.0007
DG36	N1	DC2	H41	2.8242	140.1864	0.0006
DG37	N2	DC2	O2	2.9576	143.4125	0.0002
DG36	N1	DC2	O2	2.9977	144.5145	0.0002
DG37	N2	DC1	H2"	2.8737	148.3028	0.0001
DG37	N1	DC2	O2	2.8967	142.5139	0.0001
DG36	N2	DC2	N3	2.9806	139.2300	0.0001
DC35	N4	DC2	H42	2.9822	135.0884	0.0001
DC35	N4	DG3	N1	2.9937	137.2879	0.0001
DT34	N3	DA4	H61	2.9982	135.0769	0.0001
34-37 Acceptor						
DonorResidue	Donor Atom	AcceptorResidue	AcceptorAtom	AvgDist	AvgAng	Occupancy
DG3	N2	DC35	O2	2.8319	163.5733	0.8792
DG3	N1	DC35	N3	2.9117	166.0159	0.7432
DA4	N6	DT34	O4	2.8567	163.8367	0.7263
DC2	N4	DG36	O6	2.8638	163.0148	0.7135
DC1	N4	DG37	O6	2.8641	161.1338	0.6629
DC1	N4	DG36	O6	2.8768	145.3940	0.0053
DA4	N6	DT34	H3	2.8328	137.0895	0.0017
DC2	N4	DG36	H1	2.7728	137.5835	0.0011
DC1	N4	DG37	H1	2.8136	139.9489	0.0007
DG3	N2	DC35	N3	2.9744	145.3383	0.0007
DG3	N1	DC35	O2	2.9558	139.2360	0.0003
DG3	N1	DC35	H41	2.7201	144.1630	0.0002
DC2	C1'	DG37	H22	2.8854	137.1547	0.0001
DC2	N4	DC35	N4	2.9412	140.3702	0.0001
DC2	N4	DG37	O6	2.9623	144.6618	0.0001
DC1	N4	DG37	N1	2.9917	140.9526	0.0001

Supplementary Table S4. Hydrogen bond analysis of mutant aspect 2. The values showed that all different hydrogen bonds formed among the terminal nucleotides of the MA2 were 55 and the average numbers of hydrogen bonds having occupancy higher than 45 % were 9.

1-4 Acceptor						
DonorResidue	DonorAtom	AcceptorResidue	AcceptorAtom	AvgDist	AvgAng	Occupancy
DT36	N3	DA2	N1	2.8905	165.1005	0.7405
DT34	N3	DA4	N1	2.8948	165.0554	0.7033
DC35	N4	DG3	O6	2.8680	163.2905	0.6850
DT37	N3	DA1	N1	2.8972	161.4801	0.5716
DT37	N3	DA1	O4'	2.8885	147.0318	0.0067
DT37	N3	DA1	O5'	2.8990	156.9604	0.0051
DT37	N3	DA1	N3	2.9420	161.9331	0.0041
DT37	N3	DA1	H2	2.9063	138.8059	0.0023
DT37	N3	DA1	H5'	2.8557	147.8085	0.0021
DC35	N4	DG3	H1	2.7399	136.3259	0.0012
DC35	N4	DA2	H62	2.9006	140.3036	0.0009
DT36	N3	DA2	H2	2.9448	138.8028	0.0009
DT34	N3	DA4	H2	2.9628	140.2533	0.0009
DT37	N3	DA1	H5"	2.8322	148.7928	0.0007
DT37	N3	DA1	OP3	2.8529	143.9846	0.0007
DT37	N3	DA1	OP1	2.8497	162.8973	0.0005
DT37	N3	DA2	N1	2.9716	143.3838	0.0004
DT37	N3	DA1	H61	2.9851	137.1063	0.0004
DT36	N3	DA2	H61	2.8592	140.6371	0.0003
DT37	N3	DA1	OP2	2.8990	156.3310	0.0003
DT36	C2'	DA1	H61	2.9835	145.1793	0.0003
DC35	N4	DA2	H61	2.7760	139.4010	0.0002
DC35	N4	DA2	N6	2.8584	140.5471	0.0002
DT34	N3	DA4	H61	2.8835	135.9996	0.0002
DT36	C1'	DG3	H21	2.8602	135.5089	0.0001
DT37	C4'	DA1	H61	2.9293	153.6621	0.0001
DT36	C1'	DG3	H22	2.9509	137.8074	0.0001
34-37 Acceptor						
DonorResidue	DonorAtom	AcceptorResidue	AcceptorAtom	AvgDist	AvgAng	Occupancy
DG3	N2	DC35	O2	2.8414	162.2302	0.8442
DG3	N1	DC35	N3	2.9090	165.4673	0.7507
DA4	N6	DT34	O4	2.8652	163.7184	0.6561
DA2	N6	DT36	O4	2.8759	164.0664	0.5818
DA1	N6	DT37	O4	2.8719	162.6149	0.4642
DA1	N6	DT37	O2	2.8279	159.2909	0.0108
DA1	OP3	DT37	O4	2.7962	156.6099	0.0058
DA4	N6	DT34	H3	2.8535	137.5228	0.0022
DA1	N6	DT37	O2	2.8599	150.0390	0.0017
DG3	N2	DC35	N3	2.9601	148.2027	0.0014
DA1	N6	DT37	H3	2.8438	137.7733	0.0009

Supplementary Table S5. Primers used for qPCR expression analysis. The primers were specifically design to target ORF region of each segment of FBNYV.

Segments name		Sequences	Tm°C	Product size(bp)
FBORF-R	F	TCTTCGACTTTCCACGAAGC	58	181
	R	AAAAAACGTCACGCATATACATAGACAATCC (R.C)		
FBORF-S	F	AAGGCAAGGTTGAATCTAACGG	58	170
	R	AAAAAACACTTCGACATAAACTGTATGTTCTAG(R.C)		
FBORF-M	F	TTGCGTGTTATGTTCCTGGG	58	206
	R	AAAAAACACTTCCAGTATTAGGTTGGTTT(R.C)		
FBORF-N	F	TACGAAGGTGCAAGGACATT	58	165
	R	AAAAAACTAAACCATTCTCATTAAGAACGGTTAC(R.C)		
FBORF-C	F	TGGTTTTCTTGATATGGACGACT	58	205
	R	AAAAAACAATATCCTTGTTCAAAGGAGTC(R.C)		
FBORF-U1	F	GAAGAGTCTTCTCCAGAAGAG	58	190
	R	AAAAAAGATACATCCGTATCTCTCATATGATAC(R.C)		
FBORF-U2	F	CCAACAAGGTGGTTGTTGA	58	190
	R	AAAAAAGGATTACCATAATACACTTGATAGCG(R.C)		
FBORF-U4	F	CGAGGAGGAAGAAGAACGTC	58	170
	R	AAAAAACCTATTTATTACGTCTCATTGATTGCC(R.C)		
